# Identification and molecular characterization of a novel non-specific lipid transfer protein (TdLTP2) from durum wheat

**DOI:** 10.1371/journal.pone.0266971

**Published:** 2022-04-13

**Authors:** Khawla Missaoui, Zulema Gonzalez-Klein, Sonia Jemli, Maria Garrido-Arandia, Araceli Diaz-Perales, Jaime Tome-Amat, Faiçal Brini

**Affiliations:** 1 Biotechnology and Plant Improvement Laboratory, Centre of Biotechnology of Sfax, University of Sfax, Sfax, Tunisia; 2 Departamento de Biotecnología-Biología Vegetal, Escuela Técnica Superior de Ingeniería Agronómica, Alimentaria y de Biosistemas, Universidad Politécnica de Madrid (UPM), Madrid, Spain; 3 Centro de Biotecnología y Genómica de Plantas (CBGP), Instituto Nacional de Investigación y Tecnología Agraria y Alimentaria (INIA), Universidad Politécnica de Madrid (UPM), Madrid, Spain; 4 Laboratory of Microbial Biotechnology and Enzymes Engineering, Centre of Biotechnology of Sfax, University of Sfax, Sfax, Tunisia; 5 Department of Biology, Faculty of Sciences of Sfax, University of Sfax, Sfax, Tunisia; Saint Mary’s University, CANADA

## Abstract

Non-specific lipid transfer proteins (nsLTPs) are small, cysteine-rich proteins, a part of the pathogenesis-related protein family, and numerous of them act as positive regulators during plant disease resistance, growth, and reproduction. These proteins are involved also in the intracellular transfer of lipids, as well as in plant immune responses. Besides their differences in sequences, they show similar features in their structure. However, they show distinct lipid-binding specificities signifying their various biological roles that dictate further structural study. This study reports the identification, *in silico* characterization and purification of a novel member of the nsLTP2 protein family from durum wheat, TdLTP2. It was generated and purified using the combination of gel filtration chromatography and reverse-phase high-performance liquid chromatography (RP-HPLC). Its identity was detected by sodium dodecyl sulfate polyacrylamide gel electrophoresis (SDS-PAGE) and mass spectrometry (MALDI-TOF). TdLTP2 had been expressed in different stress to detect its localization; therefore, fluor-immunolocalization studies accomplished this data. In this approach, to assess the allergenicity of TdLTP2, thirty patients with baker’s asthma were enrolled and ELISA to detect the presence of specific IgE antibodies tested their sera. Moreover, the lipid-binding properties of TdLTP2 were examined *in vitro* and validated using a molecular docking study. In summary, our results demonstrate a new addition of member in plant nsLTPs family, TdLTP2, which can develop a better understanding about its biological functions and shed light on future applications.

## 1. Introduction

Non-specific lipid transfer proteins (nsLTPs) are an important class of pathogenesis-related proteins (PR-14) involved in various physiological functions in plants. They are involved in the facilitation of the intermembrane transfer of lipids [1, 2], lipid metabolism [3, 4] and adaptation to various environmental conditions [5] like biotic and abiotic stress responses [6, 7], such as the case for the durum wheat TdLTP1 which is induced by ABA, ethylene (ET), methyl jasmonic acid (MeJA) and salicylic acid (SA) [8]. LTPs proteins were discovered firstly in 1970 and were originally named phospholipid exchange proteins, but later they were renamed phospholipid transfer proteins. Supplemental studies showed that not only phospholipids, but other hydrophobic molecules along with may be ligands of such proteins, and, consequently, nsLTPs were given their last name ‘non-specific lipid transfer proteins’ [9]. Several binding experiments *in vitro* show that nsLTPs bind both saturated and unsaturated fatty acyl chains, presented in various molecules such as in Lysophosphatidylcholine (LPC), Phosphatidylglycerol (PG), acyl-CoA or as free fatty acids [10]. Also, some of the nsLTPs are classified as allergens [3, 11–13] related to their role in transport of lipids. Do to their structure, stabilized by disulfide bonds, which is responsible for their high resistance to cleavage by digestive enzymes as well as chemical and enzymatic degradation and enables the proteins to reach the human intestine in native immunogenic form and to cause sensitization [9].

Interestingly, nsLTP2 shave revealed flexibility in the internal pocket, which make them able to bind bigger ligands with a rigid structure, such as sterols [9, 14] and some of them were marked to bind to hydroxylated acyl chains such as the case of rice LTP2 and OsLTP2.3, which bind to dehydroergosterol [15]. In addition, at the subcellular level, nsLTPs are mainly located in the secretory pathway, which also indicates that nsLTPs are secreted proteins [16]. The excision of the signal peptide allows the targeting of the mature peptide of nsLTPs to cell secretory pathway where they are exported to the apoplast. Considering these studies together with other findings exhibiting the localization of some nsLTPs in intracellular and extracellular space in tobacco, *Arabidopsis* and wheat [6, 17] different functions are suggested for the roles of nsLTP in the physiology of plants, such as their involvement in cutin synthesis [18], β-oxidation [19], and somatic embryogenesis [20, 21].

In this study, we identified a novel TdLTP2 protein from wheat (*Triticum durum* L). We performed a strict bioinformatic approach using the protein features, including pI, signal peptide, subcellular localization, gene structure, conserved motifs, and protein three-dimensional structure to characterize TdLTP2. Moreover, the expression level of TdLTP2 was analyzed under different stress conditions and confirmed with confocal studies to unveil the localization of TdLTP2 according to previous studies. Also, *in vitro* lipid binding efficiency was performed with a wide range of fatty acids. Finally, the allergenic potential of TdLTP2 was tested using sera of patients with baker’s asthma.

## 2. Methods and materials

### 2.1. Molecular cloning and *in silico* analysis of TdLTP2

#### 2.1.1. Isolation of TdLTP2 cDNA

Total RNA from durum wheat leaf was extracted using the RNeasy mini-Kit (Qiagen). To remove contaminating genomic DNA, total RNA (10 mg) was treated with DNase (Promega). DNase-treated RNA samples (20 μg) were reverse-transcribed using MMLV reverse transcriptase (Invitrogen). The reverse transcription (RT) reaction was performed at 37°C for 1 h using 2 μM oligo-dT18 primer. Two microliters of first strand cDNA were used as template for PCR amplification of the TdLTP2, using PFU as polymerase in a reaction solution prepared according to the instructions of the manufacturer (Fermentas). Primers designed were 5’–ATGGCTCGCACTGCAGCTAC- 3’ and R: 5’- TCAGTGGATCTTAGAGCAGTC-3’. After denaturation of the cDNA for 5 min at 94°C, PCR amplification consisted in 35 cycles comprising successively 30 s at 94°C, 30 s at 60°C and 90 s at 72°C, and a final extension for 5 min at 72°C was done. Amplified products were cloned using the pGEM-T Easy vector system (Promega) and successful isolation of TdLTP2 gene was confirmed by digestion and sequencing.

#### 2.1.2. Sequence analysis, alignment, and comparison of TdLTP2 with the reference TdLTP1

Open reading frames (ORFs) and amino acid sequences were deduced using ORF finder program and homology search were conducted using BLAST 2.0 program of the National Center of Biotechnology Information (NCBI). The software ClustalW (1.82) from Europe Biotechnology Information was employed for multiple sequence alignment of amino acid sequences of wheat nsLTP with other monocot nsLTPs retrieved from GenBank. Related nsLTP sequences used for multiple alignments were searched using BLASTp algorithm. TdiLTP4.1: LTP4.1-Like from *Triticum dicoccoides* (accession number: XP_037411197.1); TaLTP1: LTP1 from *Triticum aestivum* (accession number: CAH04988.1); TdLTP1: LTP1 from *Triticum turgidum* subsp. *durum* (accession number: CAH04990.1); TaLTP1: LTP1 from *Triticum aestivum* (accession number: ABF14722.1); TaLTP2: LTP2 from *Triticum aestivum* (accession number: QDQ71826.1); TdiLTPCw18: LTPCw18 from *Triticum dicoccoides* (accession number: XP_037411198.1); HvLTP4.1: LTP4.1 from *Hordeum vulgare* (accession number: KAE8788659.1); AtLTP4.1: LTP4.1-like from *Aegilops tauschii* subsp. *tauschii* (accession number: XP_020191753.1).

Patterns identification and location in studied TdLTP1 (previously isolated and analyzed by our group; gene bank accession number JF799976) and TdLTP2 (gene bank accession number MK570866) were determined by using ScanProsite tool (https://prosite.expasy.org/). Signal sequences and location of their cleavage sites were predicted through SignalIP-5.0 server (http://www.cbs.dtu.dk/services/SignalP/). ProtParam online tool (http://web.expasy.org/protparam/) was used to determine the physicochemical parameters of TdLTP1 and TdLTP2 proteins. The subcellular localization prediction was carried out by using Plant-mPLoc server available at: http://www.csbio.sjtu.edu.cn/bioinf/plant-multi/ [22].

#### 2.1.3. Construction of the phylogenetic tree

The phylogenetic tree of 25 nsLTP sequences was constructed using MEGA.X version 10.0.5 software [23]. The evolutionary history was inferred by using the Maximum Likelihood method and JTT matrix-based model [23, 24]. Initial tree(s) for the heuristic search were obtained automatically by applying Neighbor-Join and BioNJ algorithms to a matrix of pairwise distances estimated using a JTT model, and then selecting the topology with superior log likelihood value. A discrete Gamma distribution was used to model evolutionary rate differences among sites (5 categories (+G, parameter = 1.5187)). The tree is drawn to scale, with branch lengths measured in the number of substitutions per site. All positions with less than 95% site coverage were eliminated, i.e., fewer than 5% alignment gaps, missing data, and ambiguous bases were allowed at any position (partial deletion option). There was a total of 108 positions in the final dataset.

#### 2.1.4. TdLTP2-ligand complexes modeling and binding affinities calculation

TdLTP2 was modeled by homology using SWISS-MODEL server [25]. The nsLTP1 from *Oryza sativa* (PDB: 1UVC) was used as a template. Initial geometries of oleic acid and beta-sitosterol were obtained from PDB (1FE3) and HMDB (HMDB0000852) databases [26, 27], respectively.

Geometries of both complexes were determined in protein-ligand docking calculations with the AutoDock VINA method [28] using the interface of UCSF Chimera [29], developed by the Resource for Biocomputing, Visualization, and Informatics at the University of California, San Francisco. Spatial grids for the geometry search were defined to include the large internal cavity of the nsLTPs, choosing a box of 40 x 40 x 40 Å that let extra space at both ends of the protein. The pocket size cavity was calculated by DogSite method implemented in the Proteins Plus server. Three rounds of docking calculations were performed for each complex in order to select the ligand location and orientation with the lowest estimated binding affinity energy. Visualization of the structures was made using The PyMOL Molecular Graphics System, Version 2.0 Schrödinger, LLC; and Chimera.

### 2.2. Expression profile of TdLTP2

#### 2.2.1. Plant materials, growth conditions, and stress treatments

After transplanting seeds of the durum wheat cv. Om Rabiaa at 25°C in the culture chamber, waiting for its germination and growth, leaf tissues and roots were harvested after 0, 24, 48, and 72 h under different stress conditions and the control plants corresponding to each time point were treated with sterile water. For stress treatment, 10 days old wheat seedlings were treated with 500 μM salicylic acid (SA), 150 mM sodium chloride (NaCl), and 100 μM Abscisic acid (ABA). Intact tissues of different wheat organs from 10 days old seedlings were collected for tissue-specific expression analysis. All freshly collected samples were rapidly frozen in liquid nitrogen and stored at −80°C until the extraction of total RNA.

#### 2.2.2. Quantitative RT-PCR

Total RNA was extracted using the Trizol reagent (Invitrogen), and 20 μg of total RNA was used to synthesize first-strand cDNA using 2 μM oligo-dT18 primers with M-MLV reverse transcriptase (Promega, USA) according to the manufacturer’s instructions. Specific primers were designed by PRIMER3plus (software (http://primer3plus.com/cgibin/dev/primer3plus.cgi); qRT-PCR was performed using SYBR Green fluorophore (Applied Biosystems) on a Bio-Rad CFX Real-Time Thermocycler (Hercules, CA, USA) by Qin et al. [30]. Three biological experiments were repeated, and the threshold cycles (Ct) of each test target were averaged for triplicate PCR reactions. The relative expression ratio of the TdLTP2 gene was calculated by using the comparative CT method with the actin gene (actin_Fw: 5′-TCCCTCAGCACATTCCAGCAGAT-3′ and actin_Rv: 5′- AACGATTCCTGGACCTG CCTCATC-3′) as an internal expression standard [31]. The relative expression level was calculated from triplicate measurements based on the 2^−DDCT^ equation: DDCT = (CT_Tstress_ − CT_UBQ10stress_) − (CT_Tcontrol_ − CT_UBQ10control_), where CT_Tstress_ is the CT of the target gene from stressed samples, CT_UBQ10stress_ is the CT of the UBQ10 gene in stressed samples, CT_Tcontrol_ is the CT of the target gene from control samples and CT_UBQ10control_ is the CT of the UBQ10 gene in control samples.

#### 2.2.3. Statistical analyses

Microsoft Excel software was used to calculate mean values and standard errors. A one-way analysis of variance (ANOVA) was performed using the SPSS 16.0 (SPSS Inc., Chicago, Illinois, USA) statistical software to determine the significant differences between control and treatment or between time-course points. The probability (P) value < 0.05 was used to measure the significant change with unequal variance.

#### 2.2.4. Subcellular localization of TdLTPs

After transplanting seeds of the cv. Om Rabiaa at 25°C in the culture chamber, waiting for its germination and growth, leaf tissues, seeds and roots were dissected and prepared for histochemical treatment, to evaluate the presence of TdLTP2 on sections of seed, leaves and roots of wheat. Organs fragments were fixed in a solution of 4% formaldehyde in PBS under vacuum over the night at 4°C. The samples were then dehydrated in an ascending series of methanol solutions and embedded in paraffin 10 μm sections of the paraffin-embedded. Organs were cut using a microtome (Leica RM1235, Wetzlar, Germany). Slides were deparaffinated in xylene, then rehydrated in 100% ethanol for 15 min, ethanol 96%, and 70% (10 min each), 15 minutes PBS and incubated stained for 10 min with PBS Triton + DAPI. Samples were incubated with anti-LTP (provided by Carlos Pastor, Hospital Universitario Fundación Jiménez Díaz, Madrid, Spain) in PBS for 90 min, and then washed with PBS three times. Samples were revealed with secondary antibody anti-rabbit-Alexa 546, washed with PBS and kept with Prolong Gold antifade reagent (Invitrogen). Images were collected on a confocal laser scanning microscope (Zeiss LSM 880).

### 2.3. Purification and characterization of the recombinant protein (rTdLTP2)

Specific encoding cDNA of *T*. *durum* TdLTP2 (Gene Bank accession no. MK570866) was cloned via yeast expression vector pPICZαA and sequenced. Recombinant rTdLTP2 was expressed as non-tagged protein and purified as described elsewhere [32] with slight modifications. The RP-HPLC was used to purify the fraction obtained from gel filtration chromatography using the column. The elution of protein was performed at the flow rate of 1 ml/min, using a gradient of acetonitrile from 0 to 85% in 145 min, where the absorbance was measured at 214 nm. The purified intact rTdLTP2 was quantified by BCA Protein Assay Reagent (ThermoScientific) then analyzed by MALDI-TOF (UltraFlex II, Bruker Daltonics), and confirmed by Peptide mass fingerprinting in the Proteomics Unit of the Universidad Complutense de Madrid. Analysis was performed in a 4800 Plus Proteomics Analyzer MALDI-TOF/TOF mass spectrometer (Applied Biosystems, MDS Sciex, Toronto, Canada) using α-cyano-4-hydroxy-cinnamic acid matrix. Electrophoresis gel (SDS-PAGE) was carried out and the resolved proteins were electro-transferred onto a nitrocellulose membrane to visualize the protein.

### 2.4. *In vitro* lipid binding activity of rTdLTP2

The binding ability of rTdLTP2 to the previously described native ligand of Tri a14 [33] was detected using thin-layer chromatography (TLC) on silica gel–coated plate (Merck, USA) developed with ethanol: acetic acid: ethyl acetate (6:3:1) in a saturated chromatography chamber and visualized under UV light (254 nm). Hydrophobic ELISA plates were used to study the binding ability of rTdLTP2 as previously described with slight modifications [32]. The plates were coated with oleic, linoleic, Palmitic, stearic acid or B-sisterol respectively, dissolved in methanol at 5 μM for an overnight at 4°C. Once the plates were dry, rTdLTP2 was incubated at different concentrations in PBS for 3 h at 37°C. After several washing steps with PBS, anti-LTP was used against rTdLTP2, and horseradish peroxidase (HRP)-conjugated anti-rabbit secondary antibodies were used for the detection of bound protein. Absorbance values of three independent experiments were fit to a curve with non-linear regression using one site–Specific binding equation in GraphPad Prism version 6.01 for Windows (GraphPad Software, San Diego, California, USA) to calculate a kd value for each lipid-TdLTP2 binding. Furthermore, THP1-XBlue-CD14™ activation was performed to detect the immunogenic properties of rTdLTP2 and for the analysis of THP1 assays, the statistically significant differences were analyzed by GraphPad6 using Kruskal-Wallis test with Dunn’s correction for multiple comparisons. *P*-values<0.05 were considered significant for all assays. The difference in the cytokine production was analyzed using the two-way ANOVA with Bonferroni correction for multiple comparisons. *P*-values <0.05 were considered significant.

### 2.5. Patient sera and immunoglobulin binding assays

A panel of 30 Spanish patients was recruited based on a reported history of allergic reaction to wheat (baker’s asthma). Written informed consent was obtained from all patients and the ethics committees of the corresponding hospitals approved the study. ELISA was performed for all the sera to establish the presence of specific IgE able to recognize recombinant TdLTP2. IgE levels were expressed as optical density units (OD) at 450 nm. Based on the IgE levels found in normal control sera, OD values <0.04 were considered negative. The same test was performed against rTri a 14 in order to establish the correlation between both recombinant proteins. The evaluation of IgE reactivity to *Triticum durum* and *Triticum aestivum* was accomplished by means of IgE ELISA assays. Briefly, wells were coated with either recombinant rTdLTP2 or recombinant rTri a 14 at 5μg/ml in PBS for 2 h at 37°C then blocked for 60 min at room temperature and incubated with the patients’ sera (dilution 1:4 with PBS). Specific IgE binding was detected by adding the secondary antibody anti-human IgE-HRP from goat, and 1-Step™ Ultra TMB-ELISA (ThermoScientific) as reaction substrate. The enzymatic reaction was stopped after 30 min with HCl 2N and absorbance values were determined at 450 nm.

## 3. Results

### 3.1. Isolation and sequence characterization of the wheat TdLTP2

**Fig 1:**
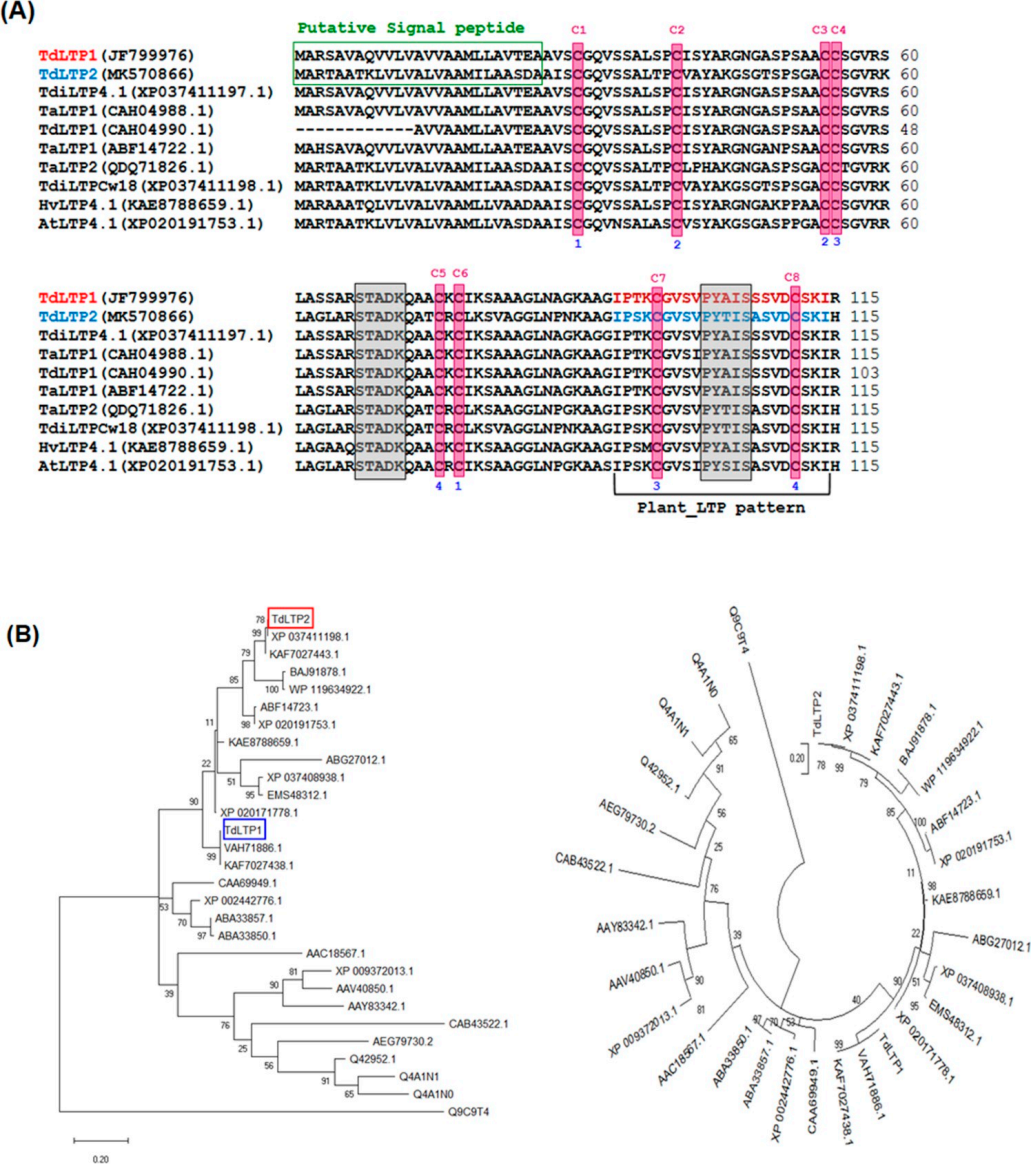
Sequence analysis of the wheat TdLTP2. Pattern and signature as revealed by ScanProsite tool with multiple alignment analysis of closed nsLTPs (A) and Molecular phylogenetic analysis of TdLTP2 sequence, with the reference TdLTP1 protein, by Maximum Likelihood method using MEGA.X program (B). The eight conserved cysteines are highlighted by magenta; the connections between the four disulfide bonds are marked by colored numbers; the two conserved pentapeptides are highlighted by grey.

The full-length cDNA of TdLTP2 was cloned and sequenced as described (see section 2). To gain insights into the occurrence of signatures, the TdLTP2 protein was firstly analyzed by ScanProsite tool using TdLTP1 as a reference. The analysis indicated the presence of an eight-cysteine motif typical of nsLTP family members as well as the existence of plant nsLTP pattern at the C-terminal. The eight conserved cysteine residues are involved in four disulfide bridges which connections are strictly conserved and are essential for functionality. For both TdLTP2 and TdLTP1, the spacing pattern within the eight-cysteine motif is the same and contains CC and CXC domains as follows: C-X_9_-C-X_14_-C-C-X_19_-C-X-C-X_19_-C-X_13_-C. However, the residue located between the cysteine numbers 5 and 6, and which is believed to play an important role in the physiological functions of nsLTPs [33], was different for both studied nsLTPs. Moreover, the physicochemical features of TdLTP2 in comparison with TdLTP1, including protein length, calculated molecular weight, theoretical pI, stability index, positive charge residues, number of cysteine and grand average of hydropathicity (GRAVY), were determined using ProtParam tool as shown in Table 1. Otherwise, SignalIP-5.0 analysis revealed that both TdLTP1 and TdLTP2 contain a putative signal peptide formed by 25 amino-acids at the N-terminal extremity (Fig 1A). Consequently, the two mature TdLTPs are consisted of 92 residues and are considered as alkaline small proteins since they have a molecular weight of about 9 kDa and a pI of about 9.1. Commonly to all known nsLTPs, no tryptophan residues are found in the sequences of TdLTP1 and TdLTP2 [34]. The plant-nsLTP pattern is sited at the C-terminal end between the residues 93 and 114 as deduced by ScanProsite analysis (Fig 1A). This pattern harbors one of the two conserved pentapeptides involved in catalytic and binding site for LTPs namely P-Y-X-I-S (residues 102 to 106). The other conserved pentapeptide T/S-X-X-D-R/K was found to be located at position 67 to 71 (Fig 1A). Phylogenetic relationships between 25 related nsLTPs sequences were inferred by the Maximum Likelihood (ML) method in MEGA.X Software. The analysis showed that TdLTP2 with the reference to TdLTP1 are located in different groups (Fig 1B). TdLTP1 is identical to the non-specific lipid-transfer protein from *Triticum turgidum* subsp. durum (accession N° VAH71886.1) and it is closely related to the type 1 nsLTP from *Triticum aestivum* (accession N° KAF7027438.1). However, TdLTP2 is identical to the non-specific lipid-transfer protein Cw18 from *Triticum* dicoccoides (accession N° XP_037411198.1) and it is closely related to the LTP2 from *Triticum aestivum* (accession N° KAF7027443.1).

**Table 1:** The physicochemical features of TdLTP2 in comparison with TdLTP1, including protein length, calculated molecular weight, theoretical pHi, stability index and grand average of hydropathicity (GRAVY), were determined using ProtParam tool.

Characteristics	TdLTP1	TdLTP2
Molecular weight of mature peptide (kDa)	9.072	9.006
Number of amino acid residues of signal peptide	21	22
Number of amino acid residues of mature peptide	94	92
Isoelectric point	9.20	9.18
Positively charged residues (Arg + Lys)	10	10
Grand average of hydropathicity (GRAVY):	0.244	0.122
number of cysteine residues and disulphide bridges	8 and 4	8 and 4
Disulphide bridges pattern	C1-C6 / C2-C3 / C4-C7 / C5-C8 (29, 77; length 49), (39, 54; length 16), (55, 97; length 42), (75, 111; length 37).	C1-C6 / C2-C3 / C4-C7 / C5-C8 (29, 77; length 49), (39, 54; length 16), (55, 97; length 42), (75, 111; length 37).
Secondary structure	Helix (3, 21; length 19), helix (29, 43; length 15), helix (52, 65; length 14), helix (71, 81; length 11), helix (87, 92; length 6), and a C-termini with a disordered region	Helix (4, 20; length 17), helix (29, 43; length 15), helix (52, 65; length 14), helix (71, 84; length 14), helix (87, 91; length 5), and a C-termini with a disordered region.
Instability index	unstable protein	Stable protein
References	[8]	This Study

### 3.2. Molecular modeling and models validation

**Fig 2:**
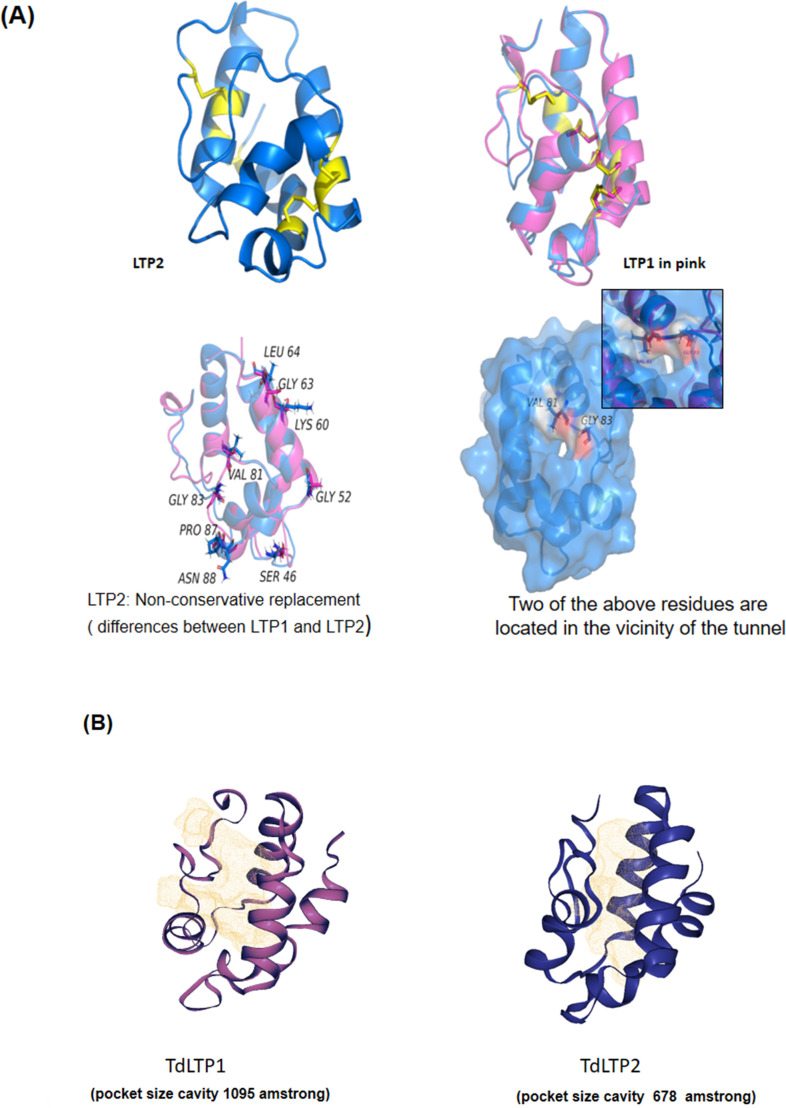
3D structural modeling of TdLTP2 with the reference model of TdLTP1. (A) Comparison of the primary and tertiary structures of TdLTP2 and TdLTP1 reveals that nsLTP subfamilies display 71% of sequence homology and share a similar α-helical fold that is stabilized by four disulfide bonds (highlighted in yellow). (B) Pocket size cavity of TdLTPs calculated by DogSite method implemented in the Proteins Plus server.

To validate the predicted models, the packing quality of each residue in TdLTP2 was evaluated using Verify 3D referred to TdLTP1. The results indicated that 100% of residues have a score indicating acceptable side chain environment and reasonably good structures. Accordingly, based on all obtained data, the predicted model of TdLTP2 was validated and retained for further investigation. The examination of the models in Fig 2 showed that the 3D structures of TdLTP2 with the reference TdLTP1 is mainly formed by four α-helices interlinked by three loops as well as the presence of a long C-terminal tail (Disulphide bonds are shown in yellow color, Fig 2). This typical structural organization was also observed in other nsLTPs from different origins [5, 33]. Besides the clear difference of pocket size cavity calculated by DogSite method implemented in the Proteins Plus server (Fig 2B), several amino acids have been outstood as mechanistically determinants and the analysis showed the different residents between both proteins which shed light to the ability of binding interaction to other molecules for TdLTP2 specifically (Fig 2).

### 3.3. Expression profile and subcellular localization of TdLTP2

#### 3.3.1. Expression profiling of TdLTP2 under different stress conditions

**Fig 3:**
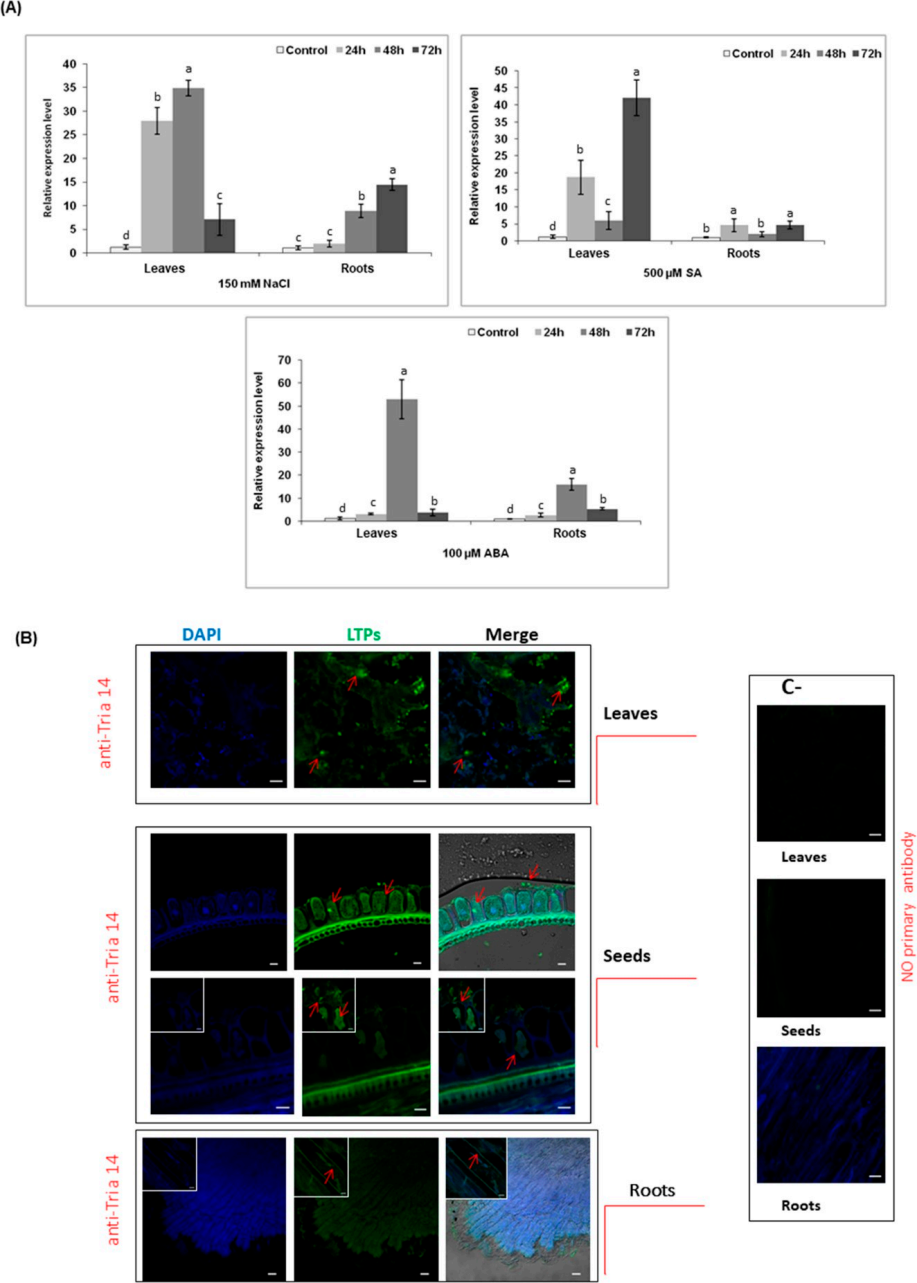
Expression profiling by QRT-PCR of TdLTP2 and immunolocalization of nsLTPs in *Triticum durum*. (A) QRT-PCR in roots and leaves tissues from durum wheat exposed to different stress conditions (150 mM NaCl, 100 μM ABA and 500 μM SA). The mock control was treated with water solution. Results were shown as means ± standard deviation of three biological replications. Relative gene quantification was calculated by the comparative Ct method. Data were normalized to the expression level of the wheat gene factor CDC (GenBank accession no. Ta54227). (B) Immunolocalization of nsLTPs sections from leaves, roots and seeds fixed with paraformaldehyde 4% and incubated with Alexa 647-labeled LTPs. LTPs detected with anti-Tri a 14 antibody (green) have been added. DAPI (blue) was used as control. Bar: 10 microns. The probability (P) value < 0.05 was used to measure the significant change with unequal variance.

We have studied the expression level of TdLTP2 cDNA in leaves and roots of durum wheat cultivars Om Rabiaa exposed to different types of stresses (150 mM NaCl, 500 μM SA and 100 μM ABA) at different time points (24 h, 48 h and 72 h). qRT-PCR analysis showed that under standard conditions, TdLTP2 expression is very low in leaves and roots (Fig 3A). When exposed to 150 mM NaCl, the expression level of TdLTP2 in leaves was differentially up regulated, especially at 24 h and 48 h. However, it was slowly up regulated in roots. For SA stress, TdLTP2 expression level did not show any differences in roots, and most induction was highlighted in leaves at 24 h and reached their maximum at 72 h (Fig 3A). Concerning ABA treatment, results showed that TdLTP2 reached the maximum expression mainly in leaves at 48h of stress (Fig 3A). The probability (P) value < 0.05 was used to measure the significant change with unequal variance in all the statistic results.

#### 3.3.2. Subcellular localization of TdLTP2

To determine the specific localization of TdLTP2, we first looked for its presence in the whole organs, then we have localized with details based on its proximity to the apoplast and the results reported by Buhot et al. [34] on the binding of nsLTP to a plasma membrane receptor. A negative control had been performed without primary antibody, confirmed our positive signals.

Confocal microscopic observations revealed that TdLTPs were detected in the cell wall, in the apoplast and in the cytoplasm as show with the green signal in the Fig 3B. Knowing that our TdLTP2 was analyzed and confirmed with the anti-Tri a 14 with high affinity, we suggest that TdLTP2 is located with high prevalence firstly in seeds than in leaves (Fig 3B). The same signal was not detected in the roots which is in accordance with previous studies (Fig 3B).

### 3.4. Production and purification of rTdLTP2

#### 3.4.1. Coomassie stain, immunoblots and MALDI-TOF analysis

**Fig 4:**
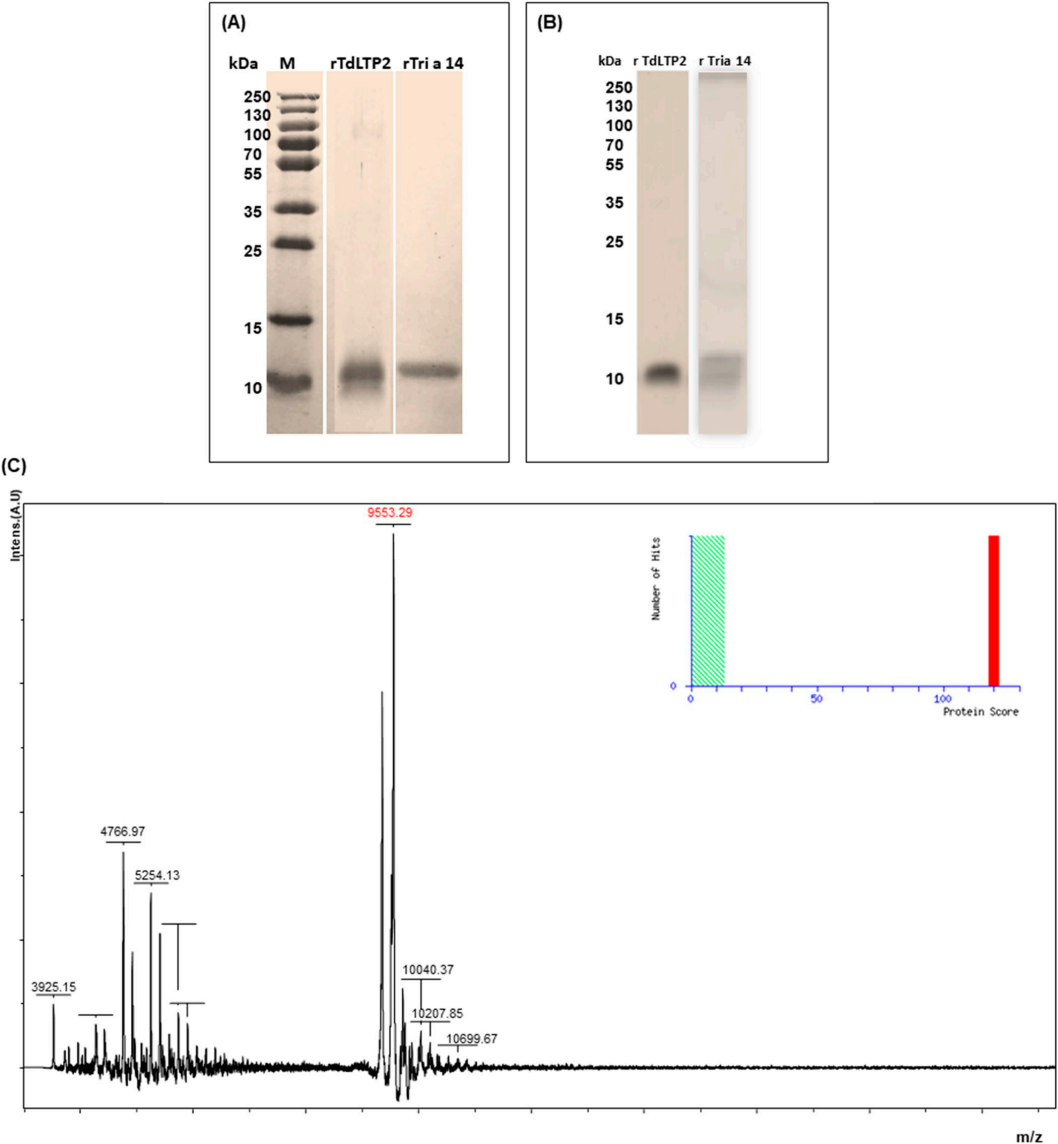
Identification and characterization of recombinant TdLTP2 (rTdLTP2). (A) Coomassie stain of rTdLTP2; (B) Western blot analysis of rTdLTP2. All analysis was performed with anti-Tria14 and rTri a 14 as a positive control. (C) MALDI-TOF mass spectra of RP-HPLC purified durum wheat rTdLTP2.

Recombinant rTdLTP2 was obtained using *Pichia pastoris*. The protein was purified by the combination of gel filtration and reverse-phase high-performance liquid chromatography (RP-HPLC). The homogeneity was determined by sodium dodecyl sulfate polyacrylamide gel electrophoresis (SDS-PAGE), then with WB as shown as in Fig 4A and 4B. Matrix-assisted laser desorption/ionization-time of flight (MALDI-TOF) mass spectrometry was used to confirm its purity and finally confirmed with Peptide mass fingerprinting (Fig 4C).

#### 3.4.2. Binding and interaction analysis of rTdLTP2

**Fig 5:**
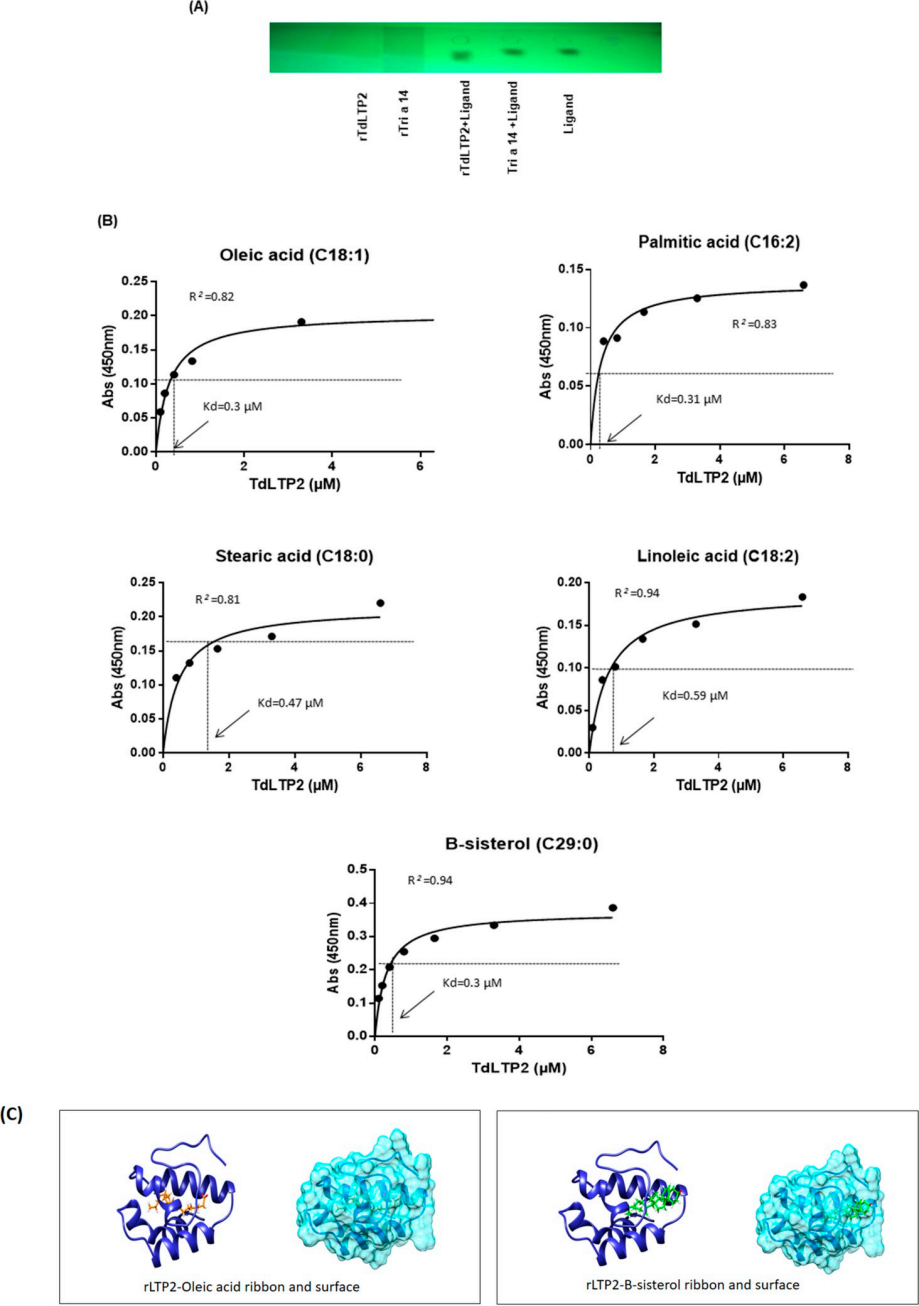
In vitro lipid binding activity of rLTP2 using different experiments. (A) Observation of the ligands by TLC in a silica gel plate and Emission of the bands under UV light (365 nm). (B) The binding ability of FAs with (50 μg/ml) for each lipid was tested at varying concentrations (6.6, 3.3, 1.6, 0.8, 0.4, 0.2 and 0.1 μM). Hydrophobic ELISA plates were coated with the indicated lipids. Protein binding was revealed using polyclonal antibody against rTdLTP2. Graphs illustrate the means of the absorbance values at 450 nm ± SEM, n = 3. (C) Molecular dynamics of oleic acid and sitosterol entering the cavity of rTdLTP2.

To understand the binding of fatty acids and sterol to rTdLTP2, three lipid binding modes were used and carried out by molecular simulation tools on the surface and inside the cavity. The result of the affinity of rTdLTP2 to the ligand of rTri a 14 performed by the thin-layer chromatography (TLC) on silica gel–coated plates (Merck, USA) was positive (Fig 5A). Hydrophobic ELISA plates were used to test the binding of rTdLTP2 with stearic acid, oleic acid, linoleic acid, palmitic acid and sterol. Results showed a high kd values specifically for oleic, palmitic acids and for sterol (Fig 5B and 5C) which is in accordance with the results of Gonzalez-Klein et al. [32].

### 3.5. rTdLTP2 displays immunity activity

**Fig 6:**
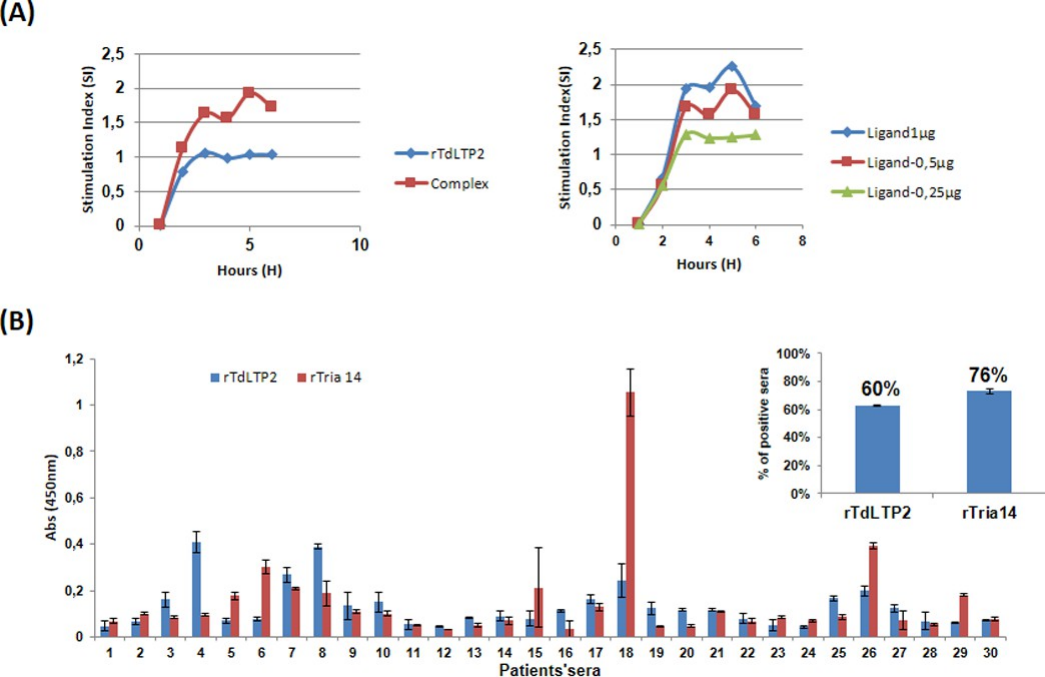
Detection of IgE patients’ sera binding to rTdLTP2. (A) Activation assay using the transfected cell line THP1-XBlue™. Cells were incubated with each ligand in triplicates and the activation was measured using QUANTI-Blue™. Results were represented by the stimulation index, referenced to the negative control (PBS), The threshold was established as the negative control +3SD. (B) Patients were sensitized to Tri a 14 with 76% (23) from a total of 30 sera. Each bar indicates the mean and SD of the inhibition percentage obtained with bakers’ sera from Spanish patients. 60% (18) sera for Tri a 14 were positives to rTdLTP2 and evaluated as significant. Threshold was calculated as the negative control+ 3SD.

As rTdLTP2 from *T*. *durum* shares 46% amino acid identity with the allergenic Tri a 14 from *T*. *aestivum* as well as 71% aa identity with TdLTP1 of *T*. *durum*, we wanted to study the immunological activity of rTdLTP2. Firstly, the immunological activity was tested using THP1-XBlue™ cells. THP1-XBlue™-MD2-CD14 cells stably express an NF-kB- and AP-1-inducible secreted embryonic alkaline phosphatase (SEAP) reporter gene. Though, upon TLR stimulation, THP1-XBlue™-MD2-CD14 cells activate transcription factors and subsequently the secretion of SEAP which is easily detectable when using QUANTI-Blue™, a medium that turns purple/blue in the presence of SEAP. In this sense, rTdLTP2 was able to induce the activation of those cells (Fig 6A), confirming its capacity to induce an immune response.

Secondly, we measured the IgE-binding capacity of TdLTP2. For this, the reactivity was determined by coating the rTdLTP2 in plate wells and incubating with sera from patients with BA (Baker’s asthma). As shown in Fig 6B, specific IgE value with a prevalence of 76% have been detected as positive for rTri a 14 and 60% from those sera have showed co-sensitization to rTdLTP2, suggesting rTdLTP2 as an allergenic protein. Our results confirmed the previous results that had been done with Safi et al. [35], which exhibited a prevalence of 69% positive to rTri a14 from sera sensitized to BA and 65% positive sera to rTdLTP1 from those sera.

## 4. Discussion

Plant LTPs comprehend a multigenic family with different genes being expressed at various stages of plant ontogeny and are believed to be involved in various physiological functions. In the present study, we isolated a new non-specific lipid transfer protein family 2 from durum wheat (TdLTP2). This one was first characterized by *in silico* analysis especially by comparing it with TdLTP1 (family 1) which had been isolated previously by our group [8]. Our results showed that the spacing pattern within the eight-cysteine motif is the same and contains CC and CXC domains as follows: C-X_9_-C-X_14_-C-C-X_19_-C-X-C-X_19_-C-X_13_-C. Interestingly, the residue X located between the cysteine numbers 5 and 6, and which is believed to play important roles in the physiological functions of nsLTPs, was different for both studied LTPs. The plant-nsLTP pattern located at the C-terminal end between the residues 93 and 114 as deduced by ScanProsite analysis, harbors one of the two conserved pentapeptides involved in catalytic and binding site for nsLTPs namely P-Y-X-I-S (residues 102 to 106). The other conserved pentapeptide T/S-X-X-D-R/K was found to be located at position 67 to 71. Otherwise, by calculating the cavities of both nsLTPs using the DogSite method implemented in the Proteins Plus server the cavity is larger in TdLTP1 than in TdLTP2 (In the case of the TdLTP2 we have found a pocket of 678 amstrong vs 1095 amstrong in the TdLTP1), which has pronounced flexibility allows accommodating bigger ligands with a rigid structure, such as sterols [36].

In this regard, the expression level of TdLTP2 was analyzed under different stress conditions and confirmed with confocal studies to unveil the localization of TdLTP2 in leaves and seeds specifically and the lack of the signal in roots which is in accordance with previous studies who showed that nsLTPs are recognized as apoplastic proteins, cell walls [37], plasma membranes [15], and to the intracellular matrix [38, 39]. Moreover, some seed nsLTPs have been detected within multiple sub-cellular localizations: intracellularly, in the cell wall, plasma membrane and in the extracellular space [38]. Furthermore, it is supposed that different biological functions of plant nsLTPs are based on their ability to bind and transport various lipid molecules. Studies of the formation of lipid binding and its stability probably depend on the hydrophobic cavity size and flexibility as well as the spatial structure of the ligand [4]. In fact, nsLTPs were originally discovered and named for their *in vitro* lipid binding or transfer ability between membranes [40, 41]. Several conserved hydrophobic amino acid residues involving Val81, Leu64, Pro87, Gly 63, Lys60, Gly52, Gly83, Asn88 and Ser46 are present in rTdLTP2. According to structural studies of nsLTPs from other plants, these hydrophobic residues are enclosed within the molecule, interacting with each other creating a suitable environment for forming a large internal tunnel-like cavity that provides a potential binding site for hydrophobic ligands [9]. The obtained data confirm that rTdLTP2 can bind a wide range of lipids as oleic, linoleic, stearic, and palmitic acids and sterol.

Different experiments have been done to test the binding ability of rTdLTP2 with lipids and sterol, confirming finally with molecular simulation that rTdLTP2 is able to bind a wide range of lipids such as oleic, linoleic, stearic, palmitic and B-sitosterol which can provide helpful data to understand their biological functions. Moreover, some studies mentioned that nsLTP2 can bind even planar sterol molecules [4], which is in accordance with our findings. In the other side, nsLTPs constitute a family of true allergens and can cause a sensitization to foods which are often characterized by thermal stability and protease resistance. Because of the extremely conserved structures, rTdLTP2 was found to be responsible for IgE cross-reactions with patients with Baker asthma with a prevalence of 60% from sera were tested with the major allergen of Baker asthma Tri a 14. This explains the presence of active allergen forms of nsLTPs in processed and heat-treated plant products, and their ability to induce both sensitization and systemic response symptoms after passing through the gastrointestinal tract [42].

## 5. Conclusion

In this study, we investigated the isolation, purification, and amino acid sequence of nsLTP2 from durum wheat (*Triticum durum*). Its 3D structure was settled *in silico* using the crystal structure. These structural analyses described TdLTP2 as it contains eight cysteine residues responsible for the disulfide bridges and a hydrophobic cavity. Interestingly, TdLTP2 was expressed mostly in leaves and concentrated in seeds, and it was confirmed by confocal studies. Moreover, *in vitro* lipid binding efficiency revealed that rTdLTP2 has the lipid-binding ability with various lipids specifically the B-sitosterol. A molecular docking study was also performed to validate the wet lab *in vitro* lipid binding study using its predicted 3D model structure. Additionally, this study reports an allergenic activity of rTdLTP2 tested and confirmed with sera sensitized with BA and performed with healthy human cells as a negative control.

## Supporting information

S1 Raw image Uncropped gels WB. (PDF)

S1 Data (ZIP)
