# Supplementary material for: Identification and molecular characterization of a novel non-specific lipid transfer protein (TdLTP2) from durum wheat
Source: PLoS One. 2022 Apr 13;17(4):e0266971. doi: 10.1371/journal.pone.0266971 (PMC9007336; doi:10.1371/journal.pone.0266971)

SDS-PAGE

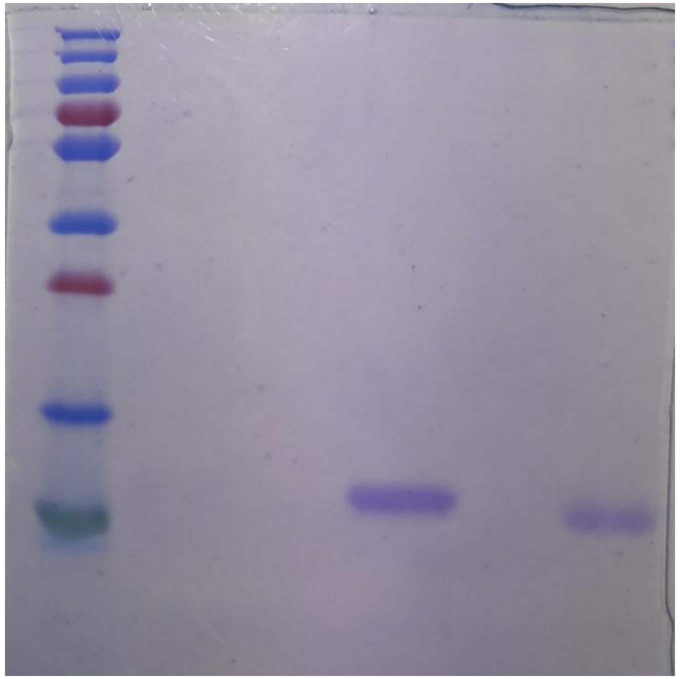

Tria 14 + Prup 3

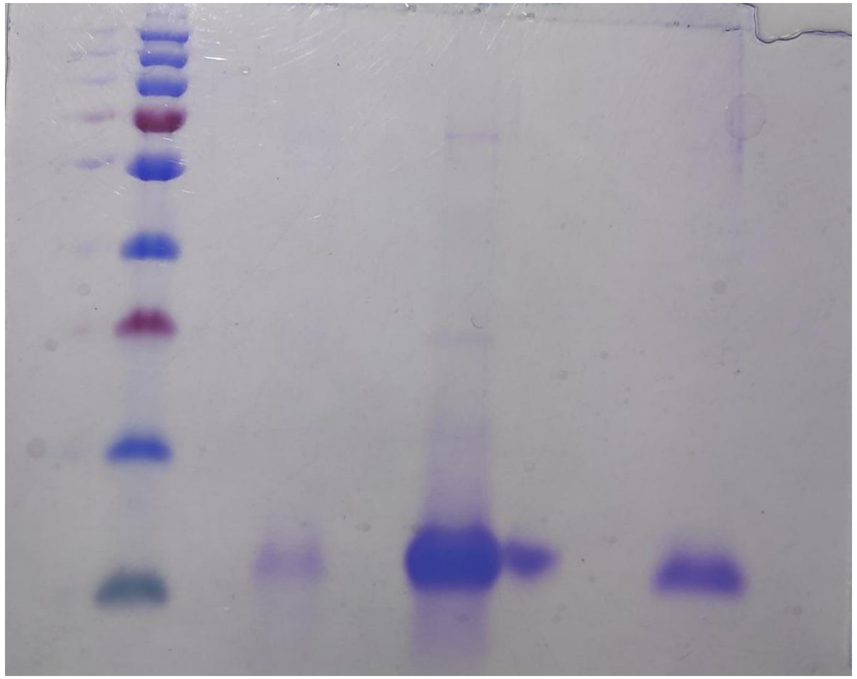

TdLTP2 (different purification)

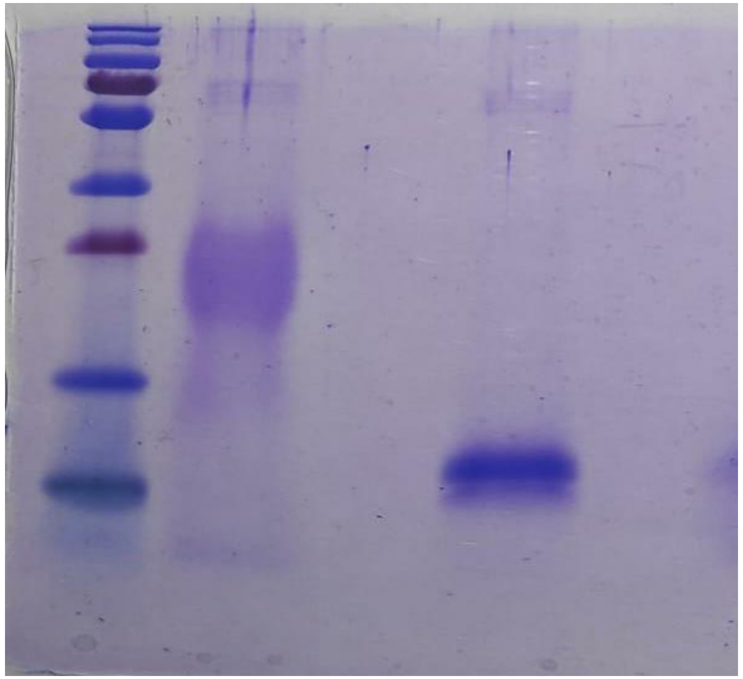

TdLTP2

Westernblot gels

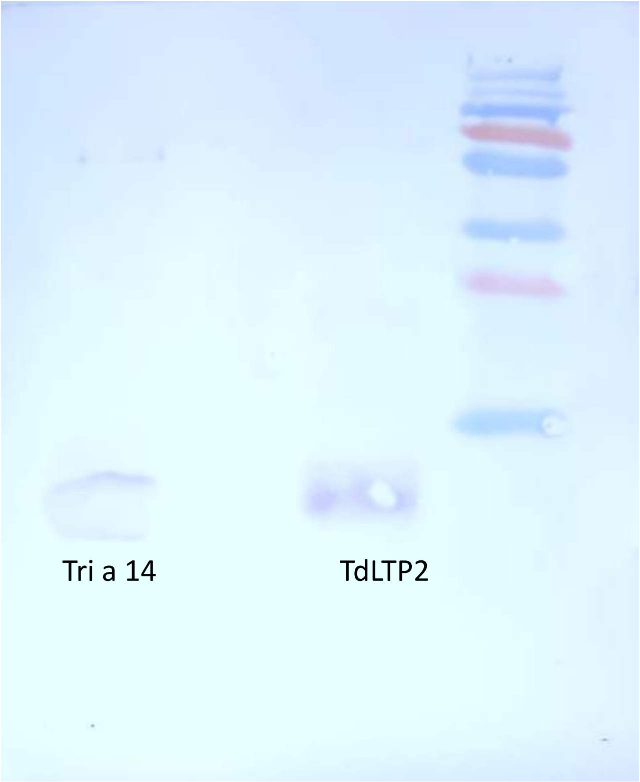

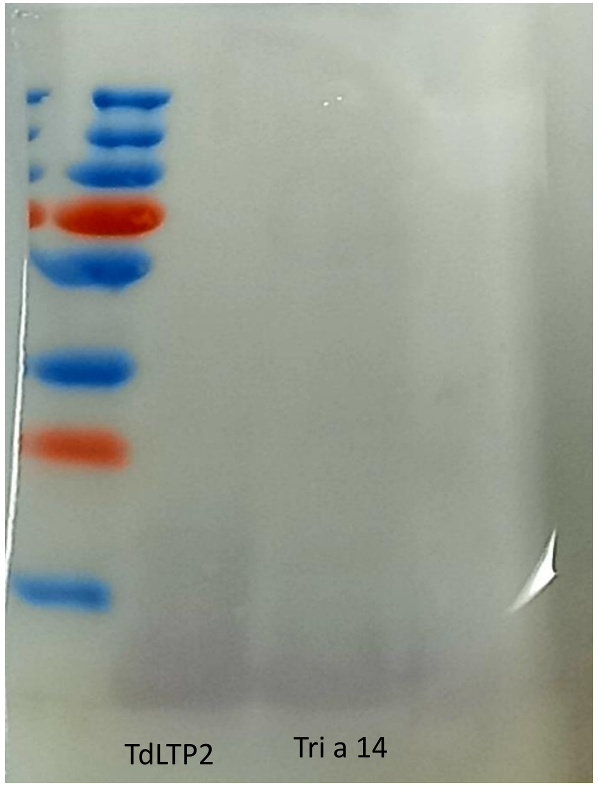

TdLTP2+Tria 14

TLC Ligand-Tria 14/TdLTP2

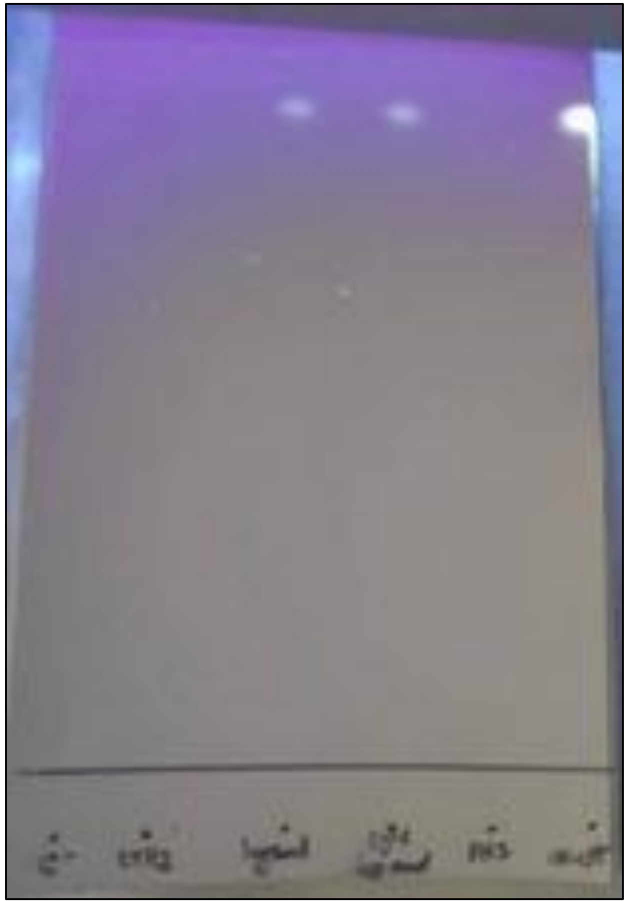

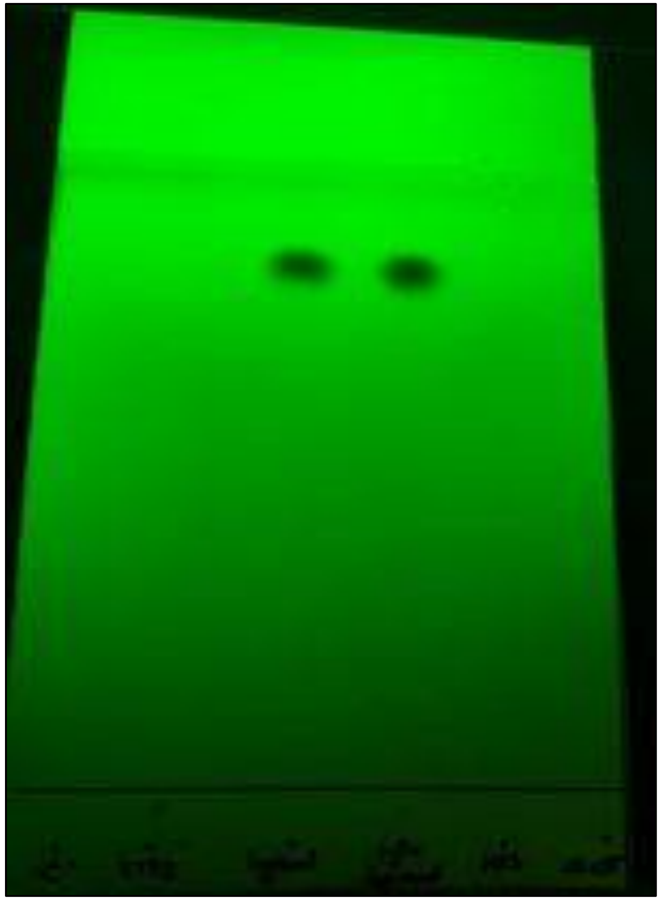

Supplement: S1 Raw image — (PDF) [file pone.0266971.s001.pdf]
