# Supplementary figures and images for: Identification and molecular characterization of a novel non-specific lipid transfer protein (TdLTP2) from durum wheat
Source: PLoS One. 2022 Apr 13;17(4):e0266971. doi: 10.1371/journal.pone.0266971 (PMC9007336; doi:10.1371/journal.pone.0266971)

## Ultraflex TOF/TOF

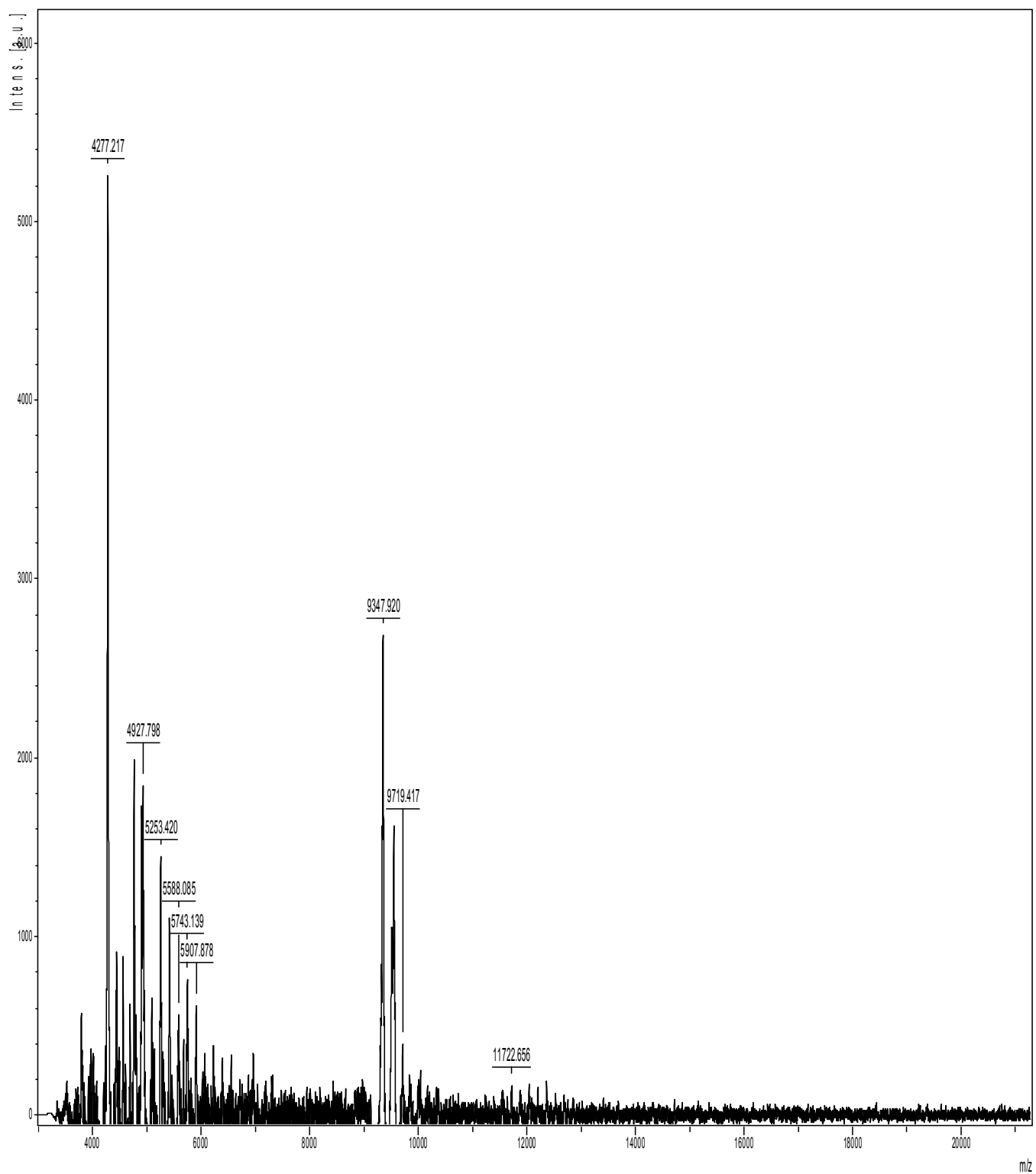

---

## Ultraflex TOF/TOF

---

| <b>m/z</b> | <b>Rel. Intens.</b> | <b>Res.</b> |
|------------|---------------------|-------------|
| 4277.217   | 100                 | 8334        |
| 4431.424   | 13                  | 705         |
| 4558.438   | 15                  | 402         |
| 4752.063   | 15                  | 1463        |
| 4927.798   | 34                  | 180         |
| 5089.912   | 11                  | 1103        |
| 5253.420   | 27                  | 266         |
| 5414.213   | 21                  | 251         |
| 5588.085   | 10                  | 253         |
| 5743.139   | 13                  | 593         |
| 5907.878   | 12                  | 302         |
| 9347.920   | 50                  | 326         |
| 9548.228   | 29                  | 272         |
| 9719.417   | 5                   | 2384        |
| 11722.656  | 3                   | 2928        |
| 11872.242  | 3                   | 4208        |
| 12031.815  | 2                   | 4441        |
| 12204.863  | 3                   | 855         |

Supplement: S1 Data — (ZIP) [file pone.0266971.s002.zip › data LTP2 paper/MALDI-TOF/LTP2-1.pdf]

## Ultraflex TOF/TOF

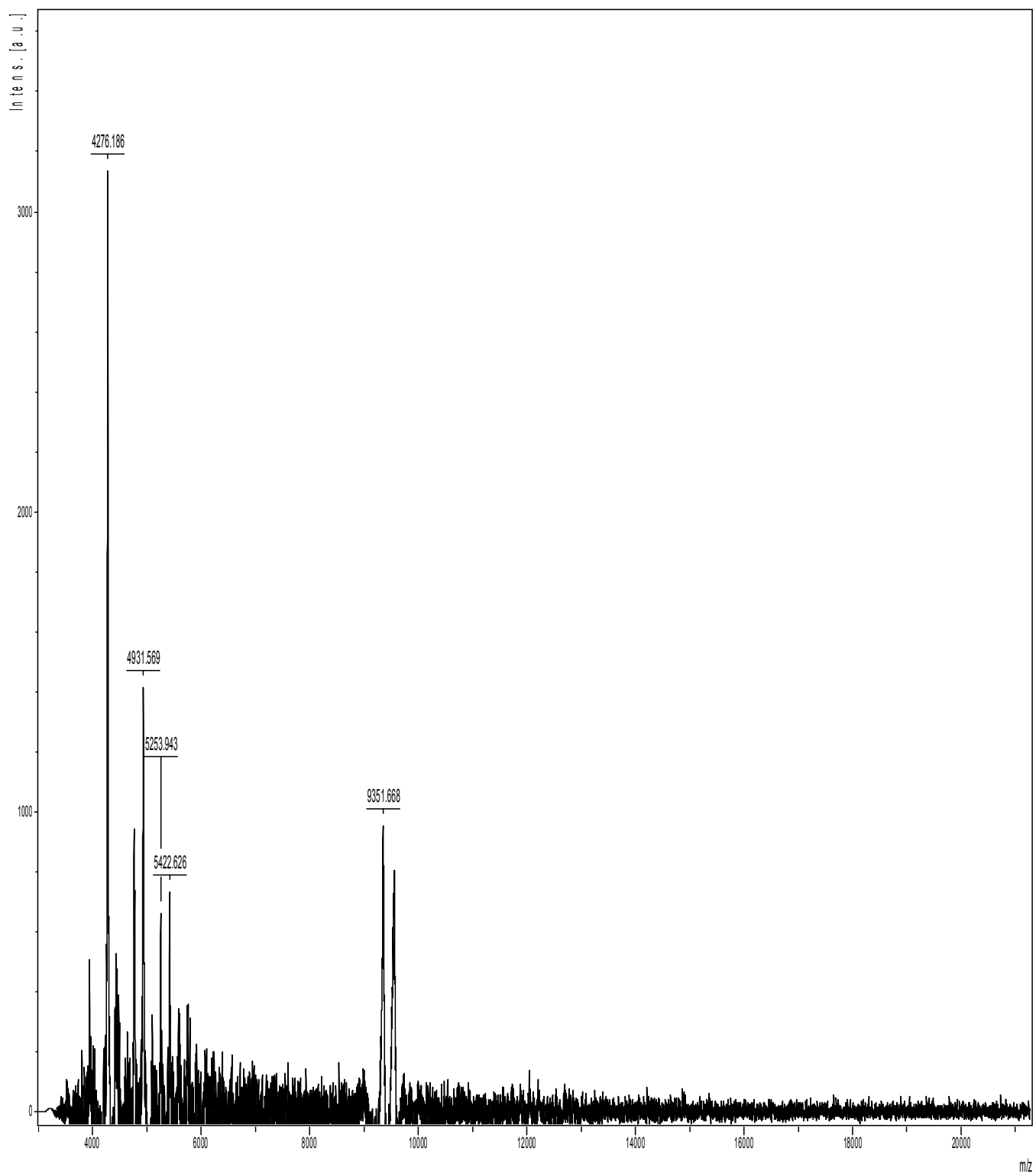

---

## Ultraflex TOF/TOF

---

| <b>m/z</b> | <b>Rel. Intens.</b> | <b>Res.</b> |
|------------|---------------------|-------------|
| 4276.186   | 100                 | 8717        |
| 4427.525   | 14                  | 1571        |
| 4761.395   | 30                  | 622         |
| 4931.569   | 44                  | 274         |
| 5253.943   | 20                  | 487         |
| 5422.626   | 23                  | 393         |
| 9351.668   | 29                  | 286         |
| 9553.189   | 24                  | 189         |

Supplement: S1 Data — (ZIP) [file pone.0266971.s002.zip › data LTP2 paper/MALDI-TOF/LTP2-5.pdf]

## Ultraflex TOF/TOF

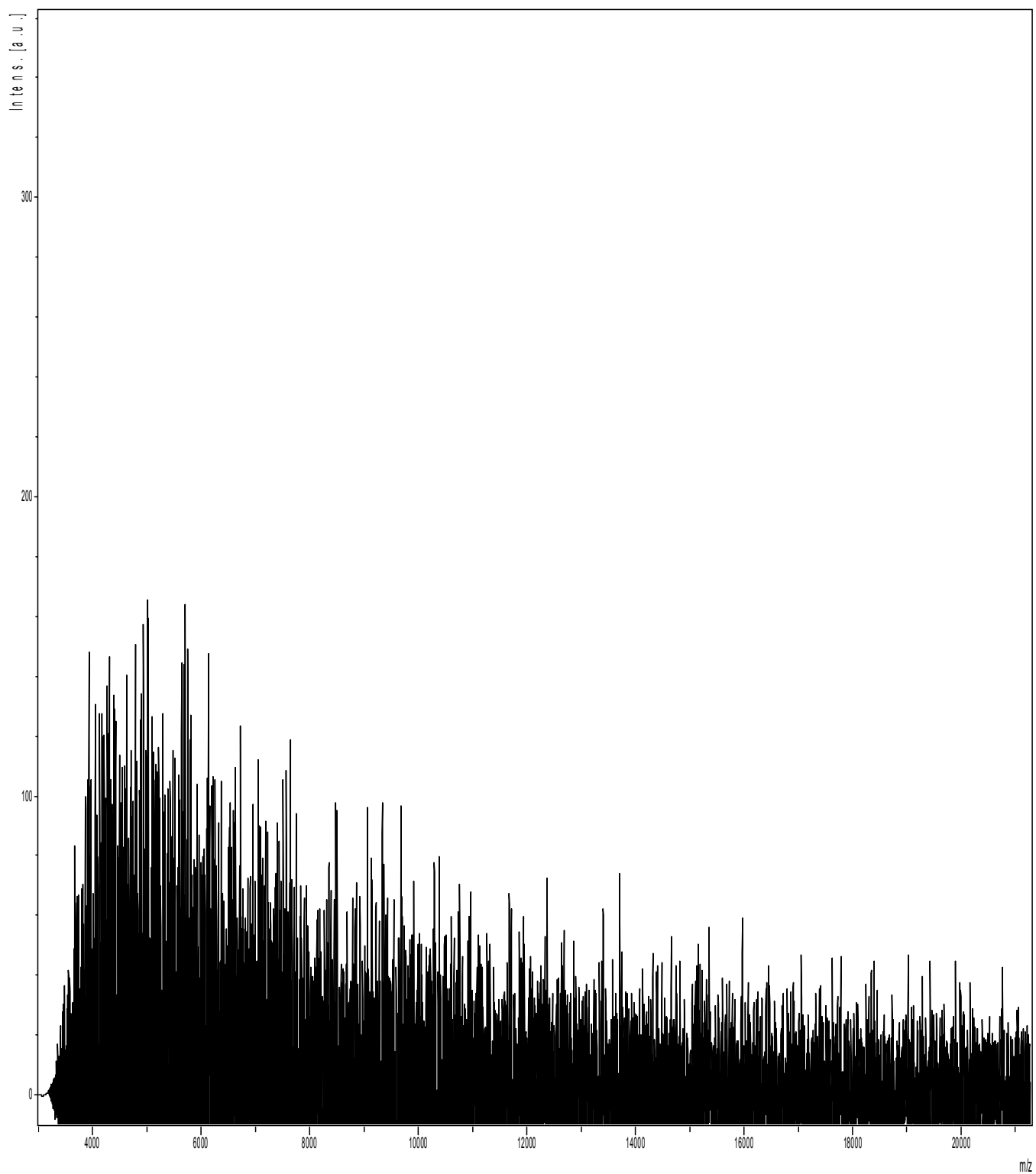

---

## Ultraflex TOF/TOF

---

| m/z | Rel. Intens. | Res. |
|-----|--------------|------|
|-----|--------------|------|

Supplement: S1 Data — (ZIP) [file pone.0266971.s002.zip › data LTP2 paper/MALDI-TOF/SUPERNATANT.pdf]
